# Activation of the SMU.1882 Transcription by CovR in *Streptococcus mutans*

**DOI:** 10.1371/journal.pone.0015528

**Published:** 2010-11-22

**Authors:** Patrick Chong, Partho Chattoraj, Indranil Biswas

**Affiliations:** Department of Microbiology, Molecular Genetics and Immunology, University of Kansas Medical Center, Kansas City, Kansas, United States of America; Tulane University, United States of America

## Abstract

In *Streptococcus mutans*, the global response regulator CovR plays an important role in biofilm formation, stress-tolerance response, and caries production. We have previously shown that CovR acts as a transcriptional repressor by binding to the upstream promoter regions of its target genes. Here, we report that *in vivo*, CovR activates the transcription of SMU.1882, which encodes a small peptide containing a double-glycine motif. We also show that SMU.1882 is transcriptionally linked to *comA* that encodes a putative ABC transporter protein. Several genes from *man* gene clusters that encode mannose phosphotranferase system flank SMU.1882 -*comA* genes. Genomic comparison with other streptococci indicates that SMU.1882 is uniquely present in *S. mutans*, while the *man* operon is conserved among all streptococci, suggesting that a genetic rearrangement might have taken place at this locus. With the use of a transcriptional reporter system and semi-quantitative RT-PCR, we demonstrated the transcriptional regulation of SMU.1882 by CovR. *In vitro* gel shift and DNase I foot-printing analyses with purified CovR suggest that CovR binds to a large region surrounding the -10 region of the P*_1882_*. Using this information and comparing with other CovR regulated promoters, we have developed a putative consensus binding sequence for CovR. Although CovR binds to P*_1882_*, in vitro experiments using purified *S. mutans* RpoD, *E. coli* RNA polymerase, and CovR did not activate transcription from this promoter. Thus, we speculate that *in vivo*, CovR may interfere with the binding of a repressor or requires a cofactor.

## Introduction

*Streptococcus mutans*, which is a part of the normal flora of the human oral cavity, is considered to be the primary etiological agent in the formation of dental caries [1], [2]. Dental caries is a global health problem that affects from 60–90% of school children, as well as many adults in industrialized countries [3], [4], [5]. Although not fatal, dental caries is a costly disease, leading to annual expenditure in billions of dollars in the U.S. alone. *S. mutans* is also a leading cause of bacterial endocarditis, with greater than 14% of viridians streptococcus-induced endocarditis triggered by infections of *S. mutans* [6]. For colonization in the oral cavity, *S. mutans* metabolizes the dietary carbohydrates ingested by its host to produce glucan and to anchor itself to the tooth surface, forming densely populated microbial biofilms known as dental plaque that includes hundreds of species of other oral bacteria [7]. The capacity of *S. mutans* to persist and to maintain a dominant presence in the oral cavity is due to its ability to adapt and respond to a variety of adverse growth-limiting conditions and production of antimicrobial peptides known as mutacins [8], [9].

Bacteria have evolved several strategies to adapt to unexpected changes in their extracellular environment, but the predominant mechanism is the so-called two-component signal transductions system (TCS) [10]. TCSs allow bacteria to coordinate a focused and appropriate physiological response to counter the effects of specific extracellular stimuli. A TCS typically consist of two main components: a membrane-bound sensor histidine kinase, which senses environmental cues, and a cytoplasmic response regulator, which upon activation by the cognate histidine kinase, elicits the appropriate cellular response, usually by binding to specific DNA target sequences, and acting as a transcriptional regulator to modulate the expression of various genes, depending on the growth condition [10].

The TCS CovRS has been extensively studied in streptococcal spp., particularly in group-A-streptococcus (GAS) [11], [12], [13], [14]. CovRS directly or indirectly regulates approximately 15% of the GAS genome, including many virulence genes. CovRS also mediates the growth of GAS during periods of iron starvation as well as growth in the bloodstream [14], [15], [16]. The vast majority of the CovR regulon is repressed by CovR [17]. CovS is thought to be the cognate sensor kinase for the phosphorylation or dephosphorylation of CovR, which typically functions as a transcriptional repressor [18]; environmental stimuli, particularly during periods of stress, are recognized by CovS, which regulates the activity of the global response regulator CovR [11]. Although CovR regulates the expression of common sets of genes in different GAS strains, there are also variations in the CovR regulon that arise between strains [19], [20]. CovRS is also associated with virulence in group-B-streptococcus (GBS) and in group-C-streptococcus (GCS) [17], [21], [22]. Approximately 6% of the genome of GBS is regulated by CovRS, including some virulence factors [22], [23]; as with GAS, the CovR regulon is mostly repressed by CovR.

*S. mutans* encodes 14 putative TCS in its genome [24], [25]; however, unlike other streptococcal spp., *S. mutans* does not encode *covS*, but does express CovR, also known as GcrR or TarC [26], [27], [28]. Therefore, CovR appears to be an orphan response regulator in *S. mutans.* As with other streptococcal spp., CovR is an important global response regulator in *S. mutans*, as it regulates the expression of various virulence genes that are essential for biofilm formation, such as the glucosyltransferases (*gtfB/C*), glucan-binding proteins C (*gbpC*), and also of its own expression [27], [29], [30].

In GAS, CovR typically functions as a transcriptional repressor [14], [17]. With respect to *S. mutans*, while CovR negatively regulates the expression of the afore-mentioned genes, our data indicates that CovR can also function as an activator of gene expression (Biswas, unpublished) one such example is an open reading frame encoded by SMU.1882. In order to gain further insight into the importance of CovR in the physiology of *S. mutans*, and the mechanism of gene regulation, we begin by having a look at the regulation of SMU.1882 by CovR. In this communication, we report that CovR, under *in vivo* conditions, activates the expression of SMU.1882. CovR directly binds to the putative promoter region of SMU.1882 and protects a region containing putative promoter elements against DNase I digestion. However, binding of CovR to the promoter of SMU.1882 did not stimulate transcription under *in vitro* conditions, suggesting other factors may be involved under *in vivo* conditions.

## Results

### SMU.1882 is unique to *S. mutans*

Bioinformatics analysis of SMU.1882 gene (354-bp) reveals that it encodes a hypothetical protein of 117 amino acid residues in length containing three double-glycine (GG) motifs, one of which is at position 23. A recent *in silico* analysis suggests that the SMU.1882 peptide is a secretory molecule [31]; the GG motif at position 23 is thought to be the site at which the cleavage occurs. However, our analyses of the genome of *S. mutans* along with other streptococcal *spp*., including *S. agalactiae*, *S. gordonii*, *S. mitis*, *S. pneumoniae*, and *S. pyogenes*, *S. thermophilus*, using software at the Comprehensive Microbial Resource website (http://cmr.jcvi.org/tigr-scripts/CMR/CmrHomePage.cgi), revealed that SMU.1882 is unique to *S. mutans*, as it is apparently not found in these streptococcal *spp*. (data not shown); however, several small GG containing peptides and homologs of the upstream SMU.1883 gene are present in some streptococci. While SMU.1882 and the adjacent *comA* homolog, SMU.1881, are unique to the *S. mutans* strains, the flanking genes, which encode mannose-phosphotransferase system (man-PTS) are very well conserved among streptococci and other *Firmicutes* [32].

### Transcriptional organization of the SMU.1882 locus

**Figure 1:**
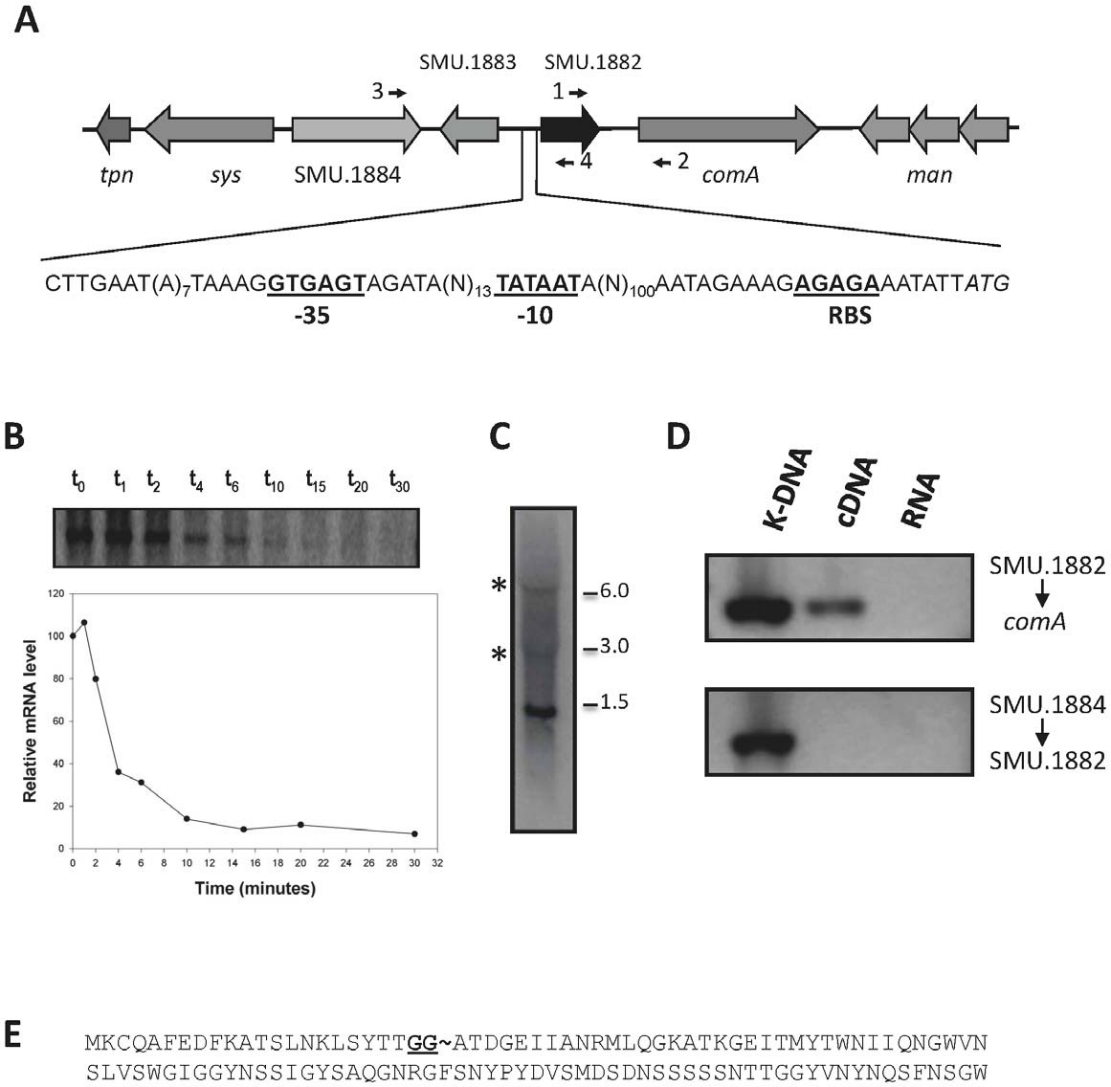
Transcriptional analysis of SMU.1882 locus. (A) Schematic diagram of SMU.1882 locus. Open reading frames (orf) are designated by block arrows, and their orientations indicate the direction of transcription. The sequence upstream of SMU.1882 orf is shown below the diagram. (B) Stability of SMU.1882 transcript. The stability of SMU.1882 transcript was measured using cultures at mid-exponential growth phase (70 KU). RNA was extracted from *S. mutans* wild type strain UA159 at the time (in minutes) indicated above the lanes following addition of rifampin to block *de novo* transcription. The mRNA decay curve was plotted as a function of time (in minutes). (C) Northern blotting was carried out using the SMU.1882 orf as a probe. A major band of 1.5 kb and two minor bands (3.0 kb and 6.0 kb) were obtained. (D) Transcriptional linkage analysis. Transcription linkage between SMU.1882/*comA*, and SMU.1884/SMU.1882 was analyzed using the primer pair 1+2 and 3+4, respectively. K-DNA denotes genomic DNA. (E) Amino acid sequence encoded by SMU.1882. The conserved GG-motif, the putative site for the signal peptide cleavage, is underlined.

A schematic diagram of the SMU.1882 locus and the genes in its vicinity are shown in Fig. 1A. Sequence analysis, confirmed using BPROM online software (Prediction of bacterial promoters, Softberry, http://linux1.softberry.com), indicates the presence of putative -35 (GTGAGT) and -10 (TATAAT) box motifs 151- and 127-bp, respectively, upstream of the putative start codon with a putative ribosomal binding site (AGAGA) 11-bp upstream of the start codon. A putative rho-independent terminator sequence (GCCTGACTACATAGATGTCAGGC) was identified at position 402-bp downstream of SMU.1882 by TransTerm (transterm.dev.java.net) program.

Since the transcripts of many CovR regulated genes have relatively shorter half-life, we therefore evaluated the stability of SMU.1882 transcript. The results of the mRNA stability assay indicated that the chemical half-life of the SMU.1882 transcript was approximately 2–3 minutes (Fig. 1B). Northern blot analysis indicated that there was one major band of 1.5-kb (which was used for the stability determination) and two other minor bands of approximately 3.0- and 6.0-kb, which constituted less than 5% of the total signal (Fig. 1C), suggesting that other genes may be cotranscribed along with SMU.1882. To determine whether the downstream *comA* (SMU.1881) was transcriptionally linked to SMU.1882, PCR analysis was performed using cDNA, with chromosomal DNA and RNA as the positive and negative controls, respectively. As shown in Fig. 1D, SMU.1882 appears to be transcriptionally linked with *comA*, which would account for the presence of the 3-kb transcript seen in the northern blot. This product could be generated due to read-through transcription from the SMU.1882 promoter.

### CovR upregulates transcription from P*_1882_ in vivo*

**Figure 2:**
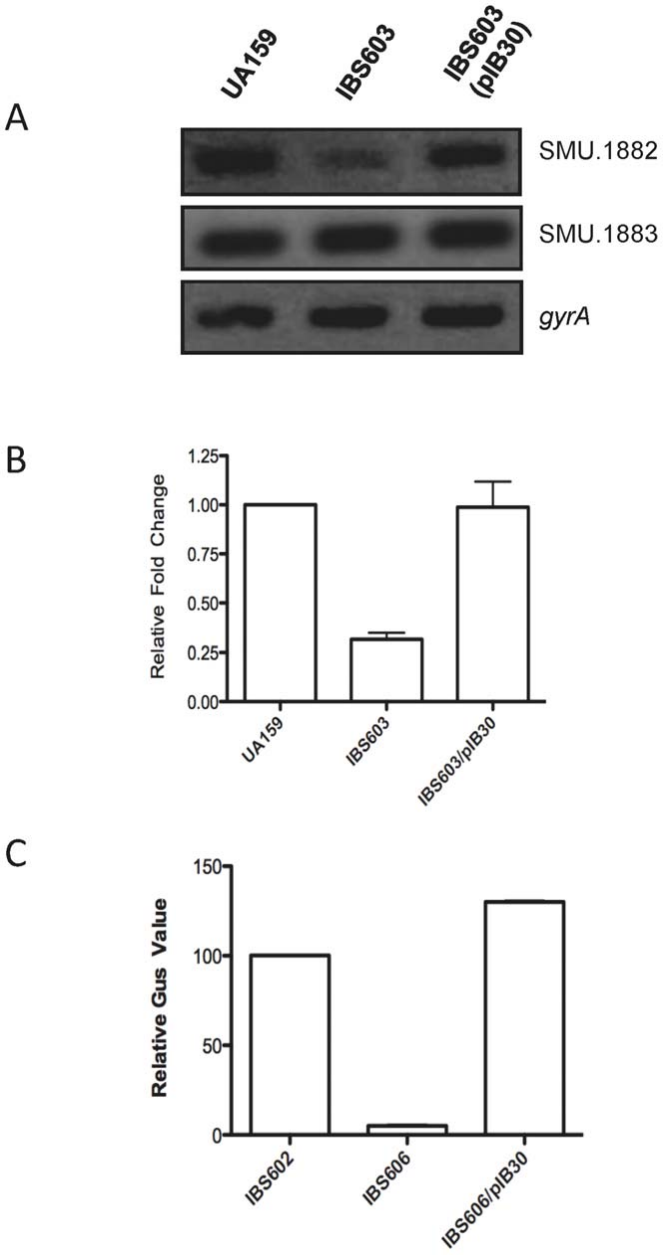
CovR is required for SMU.1882 expression. (A). Semi-quantitative RT-PCR analysis of SMU.1882, SMU.1883, and *gyrA* for the strains UA159, Δ*covR* (IBS603) and Δ*covR* complemented with pIB30 (IBS603/pIB30). The *gyrA* gene was included as an internal control to ensure that equal amounts of RNA were loaded per lane. Experiments were repeated at least twice with two independent RNA isolations. (B) Quantitative real-time PCR analysis of SMU.1882 transcript in wild type, Δ*covR* (IBS603) and Δ*covR* complemented with pIB30 (IBS603/pIB30). The expression of SMU.1882 was normalized to the expression level of *gyrA* as an internal reference. Relative fold changes in gene expression are represented as histograms with error bars. Each histogram is the mean of four biological replicates. (C) Reporter fusion assay. The putative promoter of SMU.1882 was fused to a promoter-less *gusA* reporter gene and inserted into the *S. mutans* UA159 chromosome. Expression driven from the promoters was evaluated by measuring Gus activity. Gus values were measured from at least three independent cultures; mean values with standard deviations are shown.

To gain insight into the link between CovR and expression of SMU.1882, sqRT-PCR was used to quantify the level of CovR-dependent expression of SMU.1882. Wild-type *S. mutans* UA159, and its isogenic Δ*covR* derivative, IBS603, were grown to mid-exponential phase (KU70), followed by harvesting and extraction of RNA. The *gyrA* gene was included as control to ensure that equivalent amounts of RNA were being used for each reaction. Fig. 2A shows that higher levels of SMU.1882 transcript are being produced from the wild-type strain compared to the *covR* mutant strain, with approximately three-fold difference in expression of SMU.1882; complementation of the Δ*covR* mutant strain with plasmid pIB30 (IBS603/pIB30), which carries the full length, wild-type *covR* gene in *trans*, restores expression of SMU.1882 to the wild type level. Since SMU.1883 gene appears to be divergently transcribed from the promoter upstream of the SMU.1882, we also measured the level of SMU.1883 transcript by sqRT-PCR. We found no significant difference between the wild type and the *covR* mutant strains. Thus, although SMU.1882 and SMU.1883 share the same intergenic region, CovR appears to only activate transcription of the SMU1882 gene. The results of the sqRT-PCR were further supported by real-time RT-PCR analysis. As shown in Fig. 2B, we found that the transcript level in the *covR* mutant strain was approximately 2.5-fold lower compared to the wild type strain.

To confirm that CovR can regulate SMU.1882 promoter at a non-native locus, we used a transcriptional reporter fusion for analysis to avoid any *cis*-effect that may alter the gene expression. Transcriptional fusion reporter strains were constructed by cloning the putative promoter region of SMU.1882 into plasmid pIB107, which contains a promoterless *gusA* gene [29]. This construct was inserted at the SMU.1405 locus, which is well separated from the native SMU.1882 locus [29]. When the wild type, *covR*-deletion mutant (IBS606), and complemented *covR*-deletion mutant (IBS606/pIB30) strains, containing the P*_1882_*-*gusA* transcriptional fusion reporter, were assayed for Gus activity. As shown in Fig. 2B, GusA activity was significantly reduced in the mutant strain IBS606, indicating that CovR is required to activate transcription from P*_1882_*. However, GusA activity was restored to the parental level when the Δ*covR* strain was complemented with *covR* in *trans* (IBS606/pIB30). Taken together, the results of the RT-PCR analyses and the reporter fusion assays indicate that expression of SMU.1882 is up regulated in the presence of CovR.

### CovR directly binds to P*1882 in vitro*

**Figure 3:**
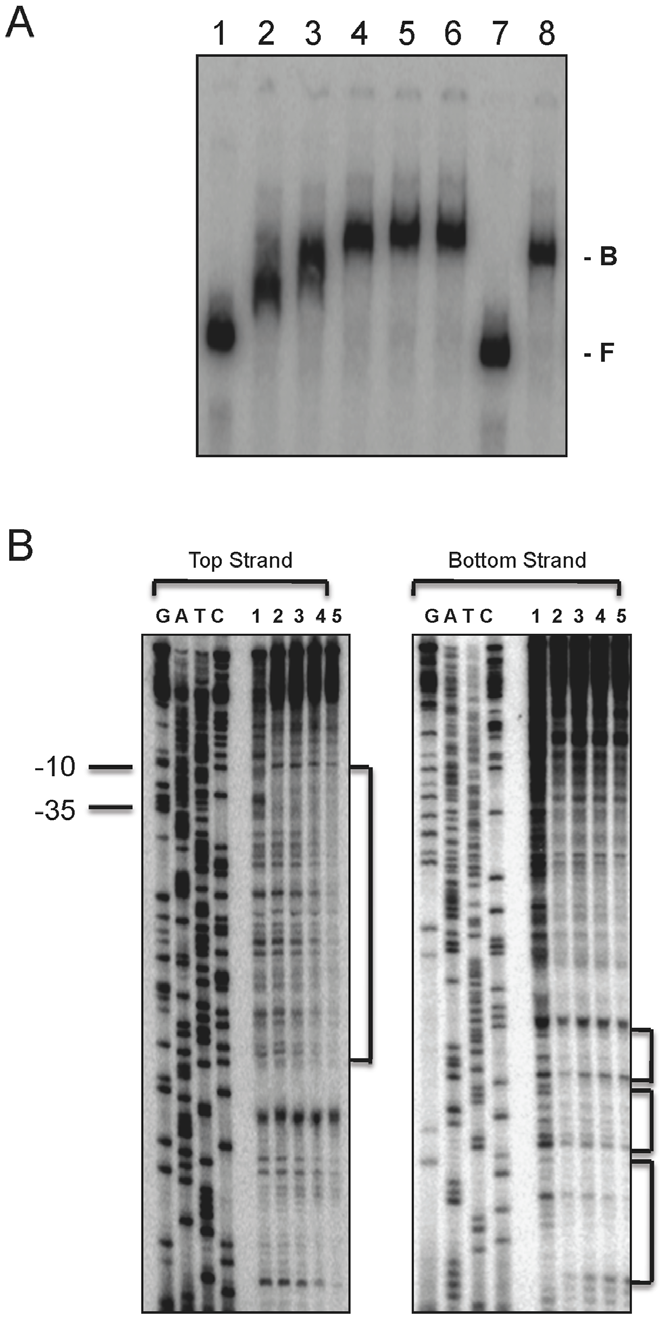
CovR binds to SMU.1882 promoter region. (A) In vitro binding of CovR to the promoter of SMU.1882. EMSA was performed with His-tagged CovR as described in the text. Lanes: 1, no CovR; 2, 14.97 pmol CovR; 3, 29.94 pmol CovR; 4, 59.88 pmol CovR; 5, 89.82 pmol CovR; 6, 119.76 pmol CovR; 7 and 8, 29.94 pmol CovR. 0.5 pmol labelled P_1882_ was used in all the reactions. 25-fold molar excess of cold P_1882_ and P_nlmA_ were used in lanes 7 and 8 respectively. B: bound DNA; F: free DNA. (B) DNase I protection assay of the putative SMU.1882 promoter. 0.5 pmol labelled P*_1882_* was used in all the reactions. Lane: 1, no CovR; 2, 59.88 pmol CovR; 3, 89.82 pmol CovR; 4, 119.76 pmol CovR; 5, 187.125 pmol CovR. Footprints were run in an 8% sequencing gel next to sequencing ladders (G, A, T, and C). CovR binding regions are indicated by vertical lines at the side of the sequencing gels, while horizontal lines mark -10 and -35 regions of the promoter. All experiments were carried out at least thrice, and a representative gel is shown.

Electrophoretic mobility shift assays (EMSA) and DNase I protection assays were used to demonstrate the specific binding of CovR to the putative promoter region of SMU.1882, P*_1882_*. As shown in Fig. 3A, incubation of the P*_1882_* DNA fragment with increasing amounts of CovR leads to retardation in the mobility of the DNA fragment. Incubation of the ^32^P-labeled P*_1882_* fragment with a 25-fold higher amount of non-labeled P*_1882_* DNA fragment resulted in no shifting of the labeled band, while the addition of a 25-fold higher amount of a DNA fragment containing the promoter region for the *nlmA* gene (P*_nlmA_*), which encodes a peptide component of mutacin IV and is not regulated by CovR, did not inhibit shifting of the CovR-bound P*_1882_* fragment (Fig. 3A). This demonstrates that CovR binds specifically to the promoter region of SMU.1882.

DNase I protection assays were performed to determine the portion of P*_1882_* that binds to CovR, using the same P*_1882_*-CovR mixtures prepared for EMSA, as described above. Analysis of the gel indicates that CovR binds to the DNA fragment from position -200 to -1, relative to the start codon, and protects this region against digestion by DNase I (Fig. 3B). This region also includes the putative ribosomal binding site, AGAGA, which is found 13-bp upstream of the start codon, as well as the putative -35 and -10 box regions. Taken together, the results of the EMSA and DNase I analyses suggest that CovR activates the expression of SMU.1882 by specifically binding to P*_1882_*.

### CovR regulation of SMU.1882 expression is not strain-specific

**Figure 4:**
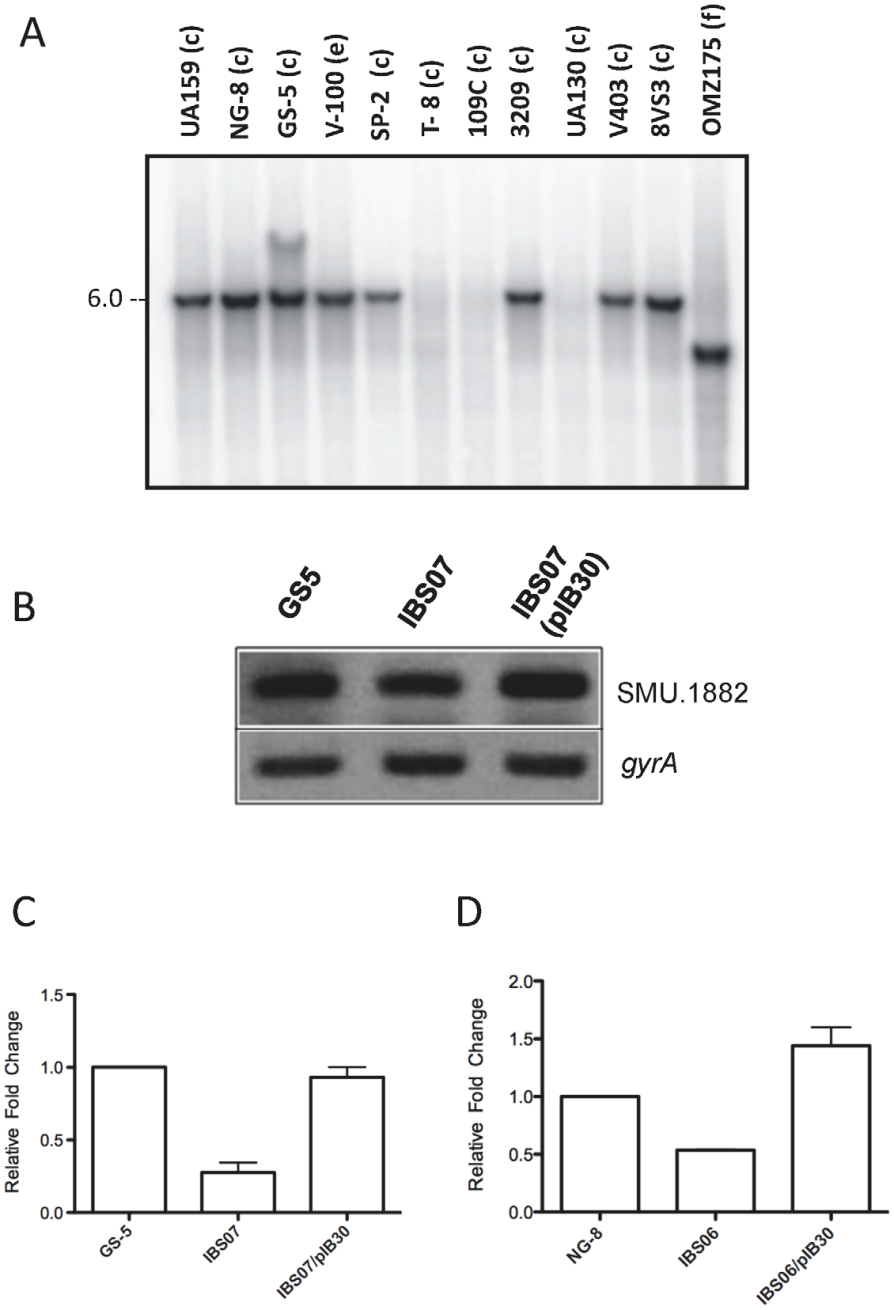
Presence of SMU.1882 locus in different strains of *S. mutans*. (A) Southern hybridization blot of *Xmn*I digested chromosomal DNA from different *S. mutans* strains belonging to three serotypes (c, e and f). A 267-bp internal SMU.1882 sequence was used as a probe against the digested DNA. Semi-quantitative RT-PCR analysis (B) and quantitative real-time PCR analysis (C) of *S. mutans* strain GS-5, its isogenic Δ*covR* strain (IBS07) and the complementing strain (IBS07/pIB30). (D) Quantitative real-time PCR analysis of SMU.1882 transcript in NG-8, its isogenic Δ*covR* strain (IBS06) and Δ*covR* complemented with pIB30 (IBS06/pIB30). The expression of SMU.1882 was normalized to the expression level of *gyrA* as an internal reference for quantitative real-time PCR analyses.

Bioinformatics analysis at the CMR website indicates that gene SMU.1882 is not found in streptococcal *spp*. outside of *S. mutans* strain UA159; however, it was not known whether SMU.1882 is conserved in other isolates of *S. mutans*. To determine whether SMU.1882 is present in other *S. mutans* strains, we used Southern hybridization to verify whether SMU.1882 was present in other isolates, from three different serotypes (c, e, and f) of *S. mutans*. A ^32^P-labeled DNA fragment containing a 267-bp internal SMU.1882 sequence derived from the genome of UA159 was used as a probe against the *Xmn*I-digested chromosomal DNA from a number of *S. mutans* isolates. The results of the Southern hybridization analysis are shown in Figure 4A. Sequence analysis predicts that, a diagnostic 6-kb DNA fragment will be generated after *Xmn*I-digestion UA159 genomic DNA. As shown in Fig. 4A, with the exception of strains T8, 109C, and UA130 that did not produce bands, the hybridization pattern observed for the majority of the isolates was identical with that of UA159 (6 kb); however, the observed band for strain OMZ175 was 4-kb. The overall results suggest that SMU.1882 is conserved in most, but not all, strains of *S. mutans*.

GS-5 and NG-8 are two other *S. mutans* strains that have been characterized to some degree. To determine whether CovR-activation of SMU.1882 is unique to strain UA159, sQRT-PCR analysis was performed on *S. mutans* strain GS-5 and its isogenic *covR* mutant derivative, IBS07; the results indicate that CovR-dependent regulation of SMU.1882 is not limited to strain UA159. Fig. 4B shows that there is a 1.8 to 2.0-fold drop in expression of SMU.1882 in IBS07 relative to the wild-type GS-5. Complementation of the *covR* mutant with full-length *covR* (IBS07/pIB30), in *trans*, restores the expression of SMU.1882 slightly higher than the wild-type level. sQ-RT results were further confirmed by real-time RT-PCR that showed a similar reduction of SMU.1882 transcript in IBS07 as compared with GS-5 (Fig. 4C). Furthermore, inactivation of *covR* in *S. mutans* strain NG8 also indicates that CovR activates the expression of SMU.1882 in this strain (Fig. 4D). Taken together, the results suggest that CovR regulation of SMU.1882 expression is not limited to the UA159 strain alone and the degree of reduction of SMU.1882 transcript in different strains appears to be similar.

### CovR does not activate expression of SMU.1882 *in vitro*

**Figure 5:**
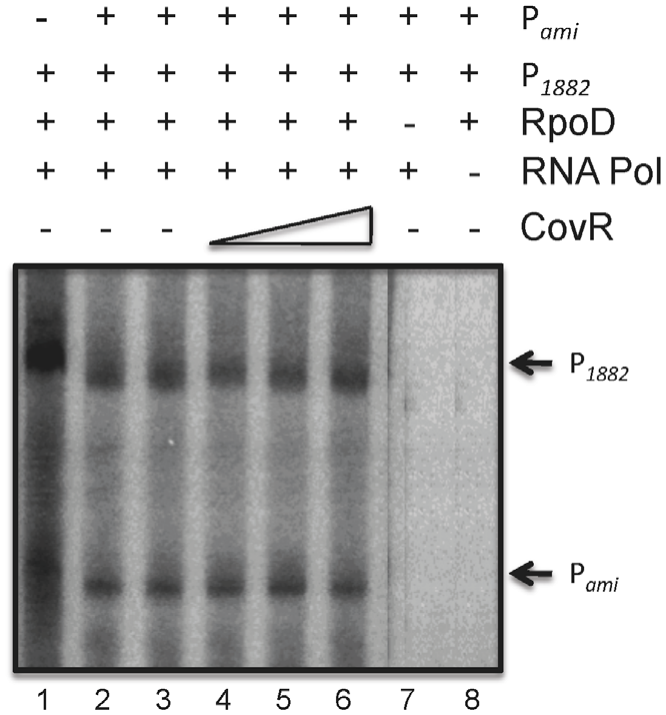
Effect of CovR on transcription from P*_1882_*. In vitro transcription was carried out using P*_1882_* and P*_ami_* template DNA according to the protocol as described in the text. Lanes 1–3: no CovR; lanes 4–6: increasing concentration of CovR (1, 4, and 10 pmole, respectively). The experiments were repeated at least twice and a representative gel is shown.

*In vitro* transcription assays were performed to demonstrate direct activation of expression from P*_1882_*. A 319-bp fragment, containing the first 82-bp of SMU.1882 as well as the putative promoter elements, was PCR-amplified from the *S. mutans* chromosome, and used as template for the *in vitro* transcription run-off assays, along with a 319-bp fragment containing P*ami* that was amplified from pIB163 which was used as a control. To perform the reaction, *E. coli* RNA polymerase core enzyme was reconstituted with purified his-tagged RpoD from *S. mutans*. As shown in Fig. 5, addition of reconstituted RNA polymerase holoenzyme, but not the core enzyme, generated expected size bands of 193-nt and 158-nt when P*_1882_* and P*_ami_*, respectively, were used as template (lanes 2 and 7). However, the addition of CovR did not lead to any observable increase in transcription from P*_1882_*. The level of transcription in the reaction mixes containing purified CovR (lanes 4–6) did not appear to be any different than the lanes that did not contain CovR (Lane 3).

## Discussion

Many bacterial TCS activate gene expression, but CovR in GAS and GBS typically acts to repress gene expression; of the 271 genes regulated by CovR in GAS, only 26 are upregulated by CovR (during late exponential phase) [17]. Our own data suggest that CovR of *S. mutans* predominantly functions as a repressor [27], [29], [30]. However, CovR can also act as a transcriptional activator in streptococci, either directly or indirectly [17], [33]; and in the case of *S. mutans*, one such gene that appears to be activated by CovR is SMU.1882. The goal of this study was to elucidate the nature of the regulatory interaction between CovR and SMU.1882.

In GAS, binding of CovR to the promoter sequences of the *has*, *covR*, and *dppA*, under *in vitro* conditions, requires the presence of a consensus binding sequence (CB) ATTARA, or the near-CB ATTAAC [33], [34], [35]. However, the importance of this sequence in the promoter regions of *S. mutans* genes for CovR-binding is unclear, since the ATTARA sequence is not found in the intragenic sequences of some CovR-regulated genes of *S. mutans* [29], [30]. Given the high degree of similarity between CovR of GAS and *S. mutans*, it is surprising that the ATTARA sequence is not more prevalent in the promoter regions of *S. mutans* genes. A single ATTAAA sequence is found 67-bp upstream of the start codon; whether this sequence is a CB for P*_1882_* remains to be studied. However, mutation of the ATTARA sites of P*dppA* in GAS did not inhibit transcription *in vivo*, although the ATTARA sequences appeared to be essential under *in vitro* conditions [33]. As such, it is possible that the lone ATTARA site in P*_1882_* may not function as a CB under *in vivo* conditions.

**Figure 6:**
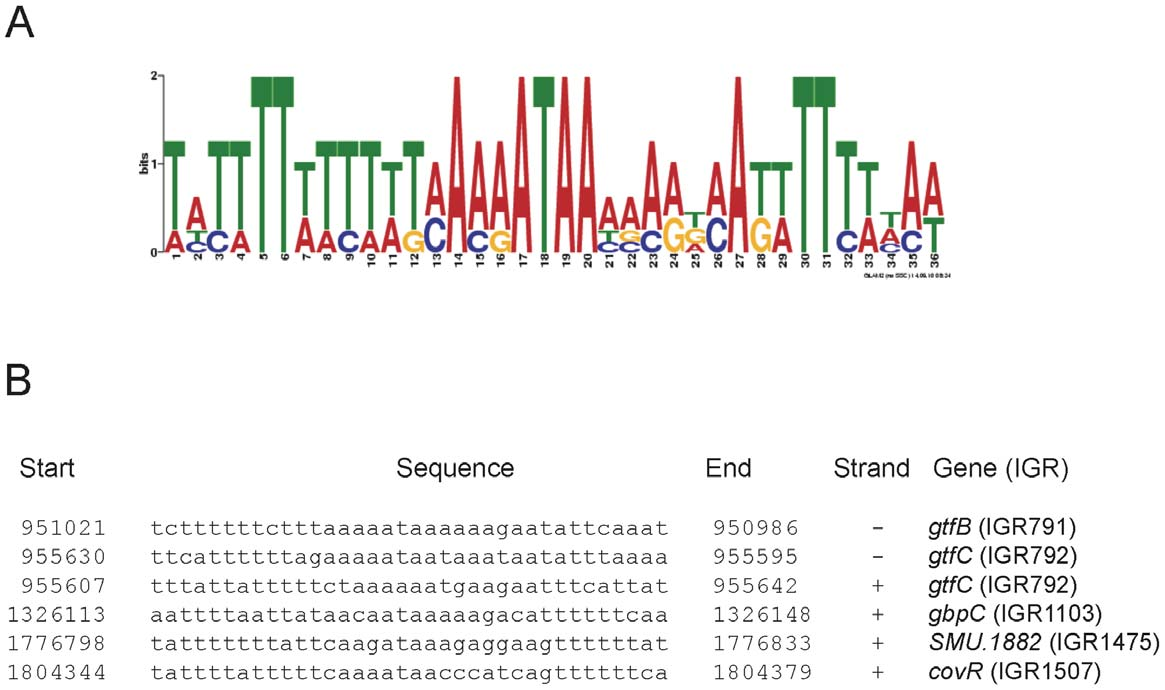
Potential consensus sequence for CovR binding. (A) WebLogo representation of the position weight matrices derived from five CovR regulated promoters using GLAM2 [36]. (B) The location of the consensus sequence in the promoter region of the corresponding genes.

In *S. mutans*, CovR has been shown to bind directly to five promoters, including P*_1882_*, to modulate gene expression [27], [29], [30]. However, a consensus DNA binding sequence (CBS) motif for CovR has not been developed. In an effort to create a CBS for CovR, we have used GLAM2 [36] program on the five promoter sequences that are regulated by CovR to develop an optimized position weight matrix for CovR binding. We obtained four different CBSs with overall weight score varied from 78.4 to 67.8. The CBS with the highest score and the location of the CBS on five promoter regions are shown in Fig. 6. Interestingly for one promoter, P*gtfC*, this CBS was present twice, one on the sense and another on the nonsense strands. However, the significance of this newly derived CBS in CovR binding needs to be verified experimentally.

The results of the sQRT-PCR and reporter expression analyses demonstrate that CovR is required for the increased expression of P*_1882_* under *in vivo* conditions, while *in vitro* gel shift and DNase I protection assays show that CovR directly binds to a portion of the intragenic region upstream of SMU.1882. Taken together, the results suggested to us that CovR might play a direct role in the activation of expression of SMU.1882. However, the results of the *in vitro* transcription assays appear to indicate otherwise, since activation of SMU.1882 expression was not observed with the addition of purified CovR. Our results are similar to the observations made with the *in vitro* transcription reactions with P*dppA* of GAS, where CovR appeared to be required for *in vivo* activation of transcription of *dppA,* but could not activate transcription under *in vitro* conditions [33]. It is always possible that the *in vitro* transcription reaction conditions were much different than the *in vivo* conditions or the role of CovR may not be to directly activate transcription of SMU.1882, but to displace other proteins that may be inhibiting or silencing transcription of SMU.1882. It is possible that the RpoD (sigma factor) does not functionally interact with *E. coli* RNA core polymerase and thus CovR's effect is not seen in vitro. However, we believe this scenario is unlikely since several heterologous sigma factors are shown to functionally interact both in vivo and in vitro with *E. coli* RNA polymerase [37], [38], [39]. Thus, how CovR activates SMU.1882 expression in vivo needs to be elucidated.

There are several possibilities by which CovR can activate gene transcription. For example, CovR can acts as an anti-silencer of gene expression. Recently, in *Salmonella*, it has been shown that response regulator such as PhoP [40] or SsrB [41] can overcome H-NS (a nucleoid-binding protein) mediated gene silencing; however, the exact molecular mechanism by which these response regulators function as anti-silencers is not yet known. However, it is conceivable that HLP, an H-NS homolog, can also silence gene expression in *S. mutans* and the role of CovR is to overcome the silencing effect of HLP by replacing it from the promoter. We believe this scenario is very unlikely since the binding of HLP to P*_1882_* is non-specific as opposed to the silenced promoters where HLP binding is specific (data not shown). Furthermore, if a repressor protein, such as HLP, binds to the intergenic region between SMU.1882 and SMU.1883, then the expression of SMU.1883 would also be affected in the *covR* deleted strain, which was not the case (Fig. 2A). The SMU.1883 gene encodes a component of the man-PTS system (ManO) and is highly conserved among various streptococci [32]. Because other genes of the man-PTS system are not regulated by CovR in other bacteria [17], we believe that this gene is not under the CovR regulatory pathway.

The other possibility is that CovR might work alongside other regulatory proteins in order to interactively regulate the expression of target genes, similar to RcsB response regulator of *Salmonella*. In this case, RcsB requires a co-activator, RcsA, for the expression of certain genes such as *ugd* [42]. On the other hand, a small molecule can bind to CovR and change its conformation/oligomerization state such that CovR can act as an activator. A well-know example of a transcriptional regulator that changes its conformation upon small molecule binding is TraR of *Agrobacterium tumefaciens*. In this case, when the cognate autoinducer ligand binds to TraR, it changes the conformation and the oligomerization of the TraR [43]. Further experiments are required to verify whether CovR interacts with other regulatory proteins or ligands as well as to understand the molecular mechanism by which CovR activates the transcription from the SMU.1882 promoter.

At the present time, the exact physiological role of SMU.1882 is unclear. This gene appears to be found only in *S. mutans*, but not in other *streptococci*. Based on the sequence and the presence of a GG motif at the N-terminal region coupled with the fact that SMU.1882 is topologically and transcriptionally associated with an ABC transporter (ComA) strongly suggest that SMU.1882 might encode a bacteriocin related peptide. Inactivation of SMU.1882 did not produce any noticeable phenotypes when tested against five streptococcal and one lactococcal species (data not shown). Analysis of the *S. mutans* UA159 genome by BAGEL [44] suggests that this strain encodes at least 26 putative bacteriocins. Since *S. mutans* resides in the dental plaque that contains over 700 different bacterial species [45], it is possible that for successful colonization and survival, *S. mutans* may need such a high number of bacteriocins. Although the target species remain to be identified, we believe that SMU.1882 is one of such bacteriocin that may provide a fitness advantage to *S. mutans* by controlling the growth of other competing species in the dental plaque.

## Materials and Methods

### Bacterial strains and growth conditions

*Escherichia coli* strain DH5α was grown in Luria-Bertani (LB) medium supplemented (when necessary) with ampicillin (50 or 100 µg/ml) or kanamycin (50 µg/ml). *S. mutans* strains UA159 and GS-5, and other mutans isolates, were typically grown in Todd-Hewitt medium (BBL, Becton Dickson) supplemented with 0.2% yeast extract (THY). Growth of the *S. mutans* cultures was monitored using a Klett-Summerson colorimeter with a red filter.

### Extraction of RNA from *S. mutans* strain UA159

RNA, which was used for various experiments, was isolated from cultures of *S. mutans* strains UA159 (and its derivatives) and GS-5 (and its derivatives), grown to mid-exponential phase (Klett unit (KU) 70), under the growth conditions specified above. The cultures were harvested *via* centrifugation, re-suspended and incubated in RNA protect reagent (Qiagen) at room temperature for 30 min, and then re-centrifuged to form a pellet, which was either stored overnight at −20°C or processed immediately for RNA extraction. RNA extraction was performed as described by Biswas *et al*. [30].

### Determination of the stability of the SMU.1882 transcript of *S. mutans*

The stability of SMU.1882 mRNA was determined using a protocol described by Biswas *et al*. [30]. Upon reaching the desired optical density (O.D.), the RNA polymerase inhibitor rifampin was immediately added to each culture to prevent the *de novo* synthesis of RNA. RNA was then collected from aliquots of *S. mutans* at 8 time points, during a total incubation period of 30 minutes at 37°C. At each time point, sodium azide (final concentration: 50 mM) was added to the culture to terminate cellular processes, and then immediately placed in dry ice. RNA (4 µg) from each time point was denatured, loaded onto a 1% agarose gel, and separated *via* electrophoresis for northern blot analysis. DNA probes were synthesized *via* PCR amplification using the primer pairs Smu1882 F3 and Smu1882 R3 (for a list of primers see Table 1), with chromosomal DNA of *S. mutans* UA159 as template. The probes were labeled using [α-32P] dATP, with a DECAprimeII kit (Ambion), and added to a hybridization tube containing the transfer membrane and ULTRAhyb buffer (Ambion), followed by overnight incubation. Following hybridization, the membrane was washed according to manufacturer's instructions, dried, and exposed to a phosphorimager plate and analyzed using a Typhoon phosphorimager (Molecular Dynamics).

**Table 1:** Primers used in the analysis.

Primer	Sequence	Function
NlmA-PF	TAAACAGCAAAAAGTAATCT	P*nlmA* amplification
NlmA-PR	GCGCATATGATAAACACCCC	P*nlmA* amplification
Smu1882F1	GCGCGGATCCTGAATAAATATATTTTATTATATC	GusA assay, EMSA, DAP
Smu1882R1	GCGCCTCAGACAGCTTATTCAAACTTGTAGC	GusA assay, EMSA, DAP
Smu1882F2	CAGCTATACTGCTGAGGCTCCCTAGATC	*In vitro* transcription
Smu1882R2	TAGCTTTAAAATCTTCAAACGCTTGAC	Linkage analysis
Smu1881cR1	CTGAAACTACAGTCAAAAGAAGATAAG	Linkage analysis
Smu1882F3	GCTGTCTTATACGACAGGTGGAGCTACTG	Southern, northern, sqRT-PCR
Smu1882R3	CCACCTGTCGTATTACTTGAACTACTGC	Southern, northern, sqRT-PCR
Smu1882F4	GCAGTAGTTCAAGTAATACGACAGGTGG	Linkage analysis
Smu1882R5	CACCATCAGTAGCTCCACCTGTCGTAT	*In vitro* transcription
Smu1883crevF1	CGCCAAGAAGAAATTTTCCCACTTTGCC	Linkage analysis
Smu1883crevR1	GGAGCCAATGTCAATCGGAAAAAGGTCAG	Linkage analysis
Smu1884cF1	GGTCAGACACCTTATTCTTAAATTGG	Linkage analysis
PJRS F	TAAGGCTATTGGTGTTTATGGC	P*ami* amplification
PJRS R	TACAATTGTCATCACCATTCTT	P*ami* amplification
Smu-Nco-RpoD-F	GGGCCATGGTAAATAATAAGAAAAAAACAT-CAAG	RpoD expression
Smu-Xho-RpoD-R	GCGCTCGAGATCTTCAACAAAATCACGTAACTGC	RpoD expression

### Semiquantitative and real-time RT-PCR analyses

Semi-quantitative (sq) RT-PCR was used to quantify the level of expression of various genes in UA159 and GS-5, along with the respective *covR* mutant strains. The *S. mutans* cultures were grown to mid-exponential phase (KU70), followed by RNA extraction. sqRT-PCR was performed as previously described [30] using the Titan one-tube RT PCR system (Roche). The *gyrA* gene was included as control to ensure that equivalent amounts of RNA were being used for each reaction. For real-time RT-PCR, cDNA was prepared from samples as described above. cDNA levels were quantified in 7500 Fast real-time PCR machine (Applied Biosystems). The reactions were performed in 20 µl final volume, consisting of 1X Fast SYBR Green Master Mix (Applied Biosystems) and 0.5 µM of each sense and antisense primer pair. For every primer pair, four different cDNA concentrations were analysed in duplicate. Cycling conditions were as follows: 95°C for 1 min, followed by 40 cycles of 95°C for 15 s and 60°C for 1 min with fluorescent measurement at the end of each cycle and one dissociation step of 95°C for 15 s, 60°C for 30 s and 95°C for 15 s. *gyrA* housekeeping gene served as an internal control to normalize the expression levels between samples. No template controls were included in all real-time PCR reactions. Standard curves were plotted (GraphPad Prism) for all three samples using log input dilution series of K-DNA templates against cycle threshold values (Ct) of the performed qPCR. Slopes and R^2^ values were obtained and were used to calculate real time PCR reaction efficiencies [E = 10̂^(−1/slope)^]. The relative expression levels were calculated according to the method developed by Pfaffl [46]. GraphPad prism software was employed to analyze the experimental data. The data are presented as mean value +/− SD.

### Construction of CovR deleted strains

To create a *covR*-deleted strain, we used pIB10 [29] that contains a 1.7-kb fragment containing the entire *covR* gene with flanking regions. A Km resistant cassette with flanking modified *loxP* sites, was amplified from pUC4ΩKm2 [47] using the primers lox71-Km-F and lox66-Km-R [48] and cloned into the *Bse*RI -*Nru*I digested and T4-DNA polymerase blunted sites to generate pIB-601. Plasmid pIB-601 was linearized with *Eco*RI and transformed into *S. mutans* UA159 as previously described [48]. Transformants were selected on THY plates containing Km; one such transformant was named IBS-601. To eliminate the *lox*P-Km^r^ cassette from the chromosome, IBS-601 was transformed with pCrePA [48], which expresses the *cre* recombinase gene from a temperature-sensitive replicon (pWV01). Subsequently, Em^r^ and Km^s^ colonies were selected; those colonies contain pCrePA and lost the Km resistance gene from the chromosome. Subsequently, pCrePA plasmid was cured as described previously to generate IBS-603, which contains a markerless *covR* deletion.

To generate *covR* deletion construct in GS-5 and NG-8, plasmid pIB26 [29] was used that contains a spectinomycin resistant gene inserted in the middle of the *covR* gene. This plasmid was linearized with *Not*I and used for *S. mutans* transformation.

### Construction of P*_1882_*-*gusA* reporter strains and quantification of transcription from P*_1882_*

To quantify transcription from P*_1882_* from *S. mutans* UA159, a reporter strain was constructed by cloning the putative promoter region of SMU.1882 into plasmid pIB107 [29], which contains a promoterless *gusA* gene. The putative P*_1882_* region was amplified from *S. mutans* chromosomal DNA using primers Smu1882 F1 and Smu1882 R1, which contain *Bam*HI and *Xho*I sites, respectively (Table 1). This fragment was digested using *BamH*I and *Xho*I, and cloned into *BamH*I-*Xho*I-digested pIB107 [29] to yield plasmid pIB602, which was then transformed into *E. coli* DH5α. Plasmid pIB602 was purified from overnight grown cultures of *E. coli* DH5α, linearized with *Bgl*I, and used for the transformation of *S. mutans* strains UA159 (wild-type), IBS603 (Δ*covR*), and IBS603/pIB30 (Δ*covR*, complemented with wild-type *covR* in *trans*) to generate the reporter strains IBS602, IBS606, and IBS606/pIB30, respectively. The reporter strains were grown to mid-exponential phase (KU70) in THY broth containing Em to maintain the complementing plasmid. The cells were harvested *via* centrifugation and lysed for β-glucuronidase (GusA) assays, as described by Biswas *et al*. [29].

### Electrophoretic mobility shift assay (EMSA) and DNase I protection assay (DPA)

EMSA and DPA were used to demonstrate binding of CovR to P*_1882_*. Primer Smu1882 F1 was labeled with γ^32^-ATP and, along with Smu1882 R1, was used to PCR amplify a DNA fragment containing P*_1882_*, with a radiolabel at the 5′-end of the fragment. Each fragment was incubated with various concentrations of 6XHis-tagged CovR protein in binding buffer (final volume - 50 µl) as described by Biswas *et al*. [30]. Following 40–45 minutes incubation, at ambient room temperature, 10 µl of the nucleo-protein mix was loaded onto a 4% (29∶1) native PAGE gel, buffered with 0.5X TBE buffer, pH 8.0. The loaded PAGE was then electrophoresed at room temperature, in the same buffer for 2 hours, 80 V. The gel was then dried and exposed to phosphorimager plate and developed using a Typhoon phosphorimager.

DPA was performed using the same P*_1882_* fragment with or without CovR. Reactions were prepared similarly to EMSA and after 40 min incubation, the mixtures were treated with a dilute DNase I solution (Epicentre) for 5 minutes, as described previously [30]. The samples were denatured and loaded onto an 8% denaturing PAGE gel containing urea and buffered with 1X TBE for electrophoresis. The gel was then dried and exposed to a phosphorimager plate.

### Purification of His-RpoD

To synthesize His-RpoD, an 1127-bp DNA fragment, containing the entire *rpoD* gene without its stop codon, was PCR amplified from chromosomal DNA of *S. mutans* UA159, using the primer pair Smu-Nco-RpoD-F and Smu-Xho-RpoD-R. The DNA fragment was digested with *Xho*I and *Nco*I, and cloned into *Xho*I/*Nco*I-digested pET23D (Novagen), generating pIB611. Plasmid pIB611 was then transformed into *E. coli* BL21 DE3 pLysS. His-RopD was purified on Ni NTA column (Qiagen) following manufacturer's protocol and the purified protein was dialyzed against buffer B.

### *In vitro* transcription (IVT) assay

A DNA fragment containing P*_1882_* was amplified from UA159 genomic DNA by PCR using the primer pair: smu1882-F2 and smu1882- R5. Similarly, the promoter P*_ami_*, which was originally isolated from a *Streptococcus pneumoniae* gene that encodes amidase, was amplified from pIB163 [49]using the primer pair: PJRS-F and PJRS-R. *In vitro* transcription assay was carried out with the desired templates in reaction buffer containing 50 mM Tris-Cl (pH 7.5), 150 mM NaCl, 2 mM MgCl2, 0.1 mM EDTA, and 0.1 mM DTT in 20 µl volume. CovR (1–10 pmol) was preincubated in the reaction buffer along with the template DNAs for 20 min at room temperature. After incubation, 500 µM each (final conc.) of ATP, CTP, GTP, and 50 µM UTP with 1.0 µCi of [α-^32^P] UTP [∼9000 Ci/mmol] were added to the reaction mixture. To initiate transcription, 0.25 U E. coli core RNA polymerase (Epicentre), RpoD (1.0 µg), and RNase inhibitor (40 U, Promega). After the incubation at 37°C for 15 min, samples were resolved by electrophoresis in a 4% urea gel. Transcripts were quantitated by densitometry with the ImageQuant software (GE Healthcare) by exposing the dried gel on a phosphorimager plate.
